# *Clock* gene is associated with individual variation in the activation of reproductive endocrine and behavior of Asian short toed lark

**DOI:** 10.1038/s41598-017-15064-7

**Published:** 2017-11-03

**Authors:** Shuping Zhang, Xianglong Xu, Weiwei Wang, Wenyu Yang, Wei Liang

**Affiliations:** 1 College of life and environment sciences, Minzu university of China, Beijing, 100081 China; 2 Ministry of education key laboratory for tropical animal and plant ecology, College of life sciences, Hainan normal university, Haikou, 571158 China; 3 Guangdong institute of applied biological resources, Guangzhou, 510260 China

## Abstract

Within year individual variation in the timing of seasonal reproduction within wild bird populations in highly seasonal environments can be pronounced, but the molecular and physiological mechanisms responsible for this variation are unclear. We investigated the relationship between *Clock* gene poly-Q length, activation of the HPG endocrine axis, and the timing of breeding behavior, in a wild population of the Asian short-toed lark (*Calandrella cheleensis*) in Inner Mongolia, China. Six variants of *Clock* gene poly-Q alleles were identified in this population. *Clock* poly-Q mean allele length was positively correlated with the mean peak date deviation of individual birds. The shorter an individual’s *Clock* poly-Q mean allele length, the earlier its plasma LH, T and E2 values peaked. Mean *Clock* poly-Q allele length of nestlings in the same nest were positively correlated with the standardized laying date of the first egg in that nest. These results suggest that the *Clock* gene influences the reproductive timing of birds through its effect on the HPG endocrine axis, and that individual variation in the timing of reproduction may have a genetic basis.

## Introduction

Seasonal timing of breeding is a nearly ubiquitous feature of birds in mid to high latitude areas. Proper timing of breeding is a key component of fitness because birds must synchronize hatching within a narrow time window of maximal food abundance to enhance their reproductive success. However, within year individual variation in the timing of reproduction within populations inhabiting highly seasonal environments can be considerable^1–5^. Although some quantitative genetic analyses have demonstrated that part of this variation has a genetic basis^6–10^, the biological mechanism of such individual variation is still not very clear. Recent studies have highlighted some candidate genes in which STR length polymorphism is associated with phenological traits^11–15^. The most commonly studied gene so far is the *Circadian Locomotor Output Cycles Kaput* (CLOCK) gene, a highly conserved transcription factor central to the rhythmicity of the circadian oscillator^16^. The CLOCK possesses a poly-glutamine (poly-Q) binding region that is polymorphic in length (the number of Q repeats), which affects its binding affinity to its transcription factor^17^. The *Clock* gene was first identified in mice and has subsequently been characterized in a large number of animals, including several bird species^18,19^. The *Clock* gene poly-Q region has been found to be polymorphic in 65 of 69 passerine populations of 12 species^18,20–25^. At the individual level, length polymorphism in the *Clock* gene poly-Q region has been found to be positively correlated with laying date, hatching date, and incubation duration, in blue tits (*Cyanistes caeruleus*)^26^, and with laying date in barn swallows (*Hirundo rustica*)^23^. Therefore, it seems quite likely that genetic variation in the *Clock* gene is involved in the within year individual variation of seasonal breeding rhythm within bird populations.

Birds in mid to high latitude areas use photoperiod as a reliable cue to activate hypothalamus –pituitary-gonad (HPG) axis endocrine to adjust the timing of their breeding behavior^27–30^. In spring, an increase in day length leads to elevated secretion of gonadotropin releasing hormone (GnRH) from the median eminence at the base of the hypothalamus^31^. This stimulates the synthesis and release of luteinizing hormone (LH) and follicle stimulating hormone (FSH) in the pituitary^32^. These two hormones are secreted into the blood and induce gonadal maturation, which, in turn, secrete testosterone (T) and estradiol (E_2_) to initiate reproductive behavior^33,34^. Although some studies imply that within year individual variation in the timing of breeding within populations could be due to genetic variation^23,26^, the relationship between the *Clock* gene and activation of the hypothalamus–pituitary–gonad (HPG) axis remains unclear. Research on rats (*Rattus rattus*)^35^ suggests that this relationship is the key to explaining the mechanism by which the *Clock* gene controls the timing of the breeding behavior. The results of this study suggested that the *Clock* gene influences the timing of reproduction in rats by binding to E-box elements in the promoter of the GnRH receptor, a key reproductive gene. This suggests that individual differences in the timing of breeding behavior could be because individuals have different versions of the *Clock* gene and consequently different HPG axis activation rates. Determining the relationship between *Clock* gene poly-Q region length and activation of the HPG axis in birds is an important test of the generality of this hypothesis, and for understanding the underlying physiological mechanism responsible for individual variation in the timing of breeding behavior.

In this paper we present data on the relationship between *Clock* gene poly-Q (hereafter ClkpolyQ) length, the activation of HPG endocrine axis, and the timing of breeding behavior in a wild population of the Asian short-toed lark (*Calandrella cheleensis*, Passeriformes, Alaudidae) in Inner Mongolia, China, a population that displays significant within year individual variation in the timing of breeding behavior^36^. Our objective was to investigate the physiological mechanism through which the *Clock* gene influences the timing of breeding of individuals in this population. Previous research conducted by us on this population indicates that it only breeds once a year, and that there is considerable variation between pairs in the dates on which the first eggs are laid and the first chicks are hatched; the earliest and latest laying and hatching dates are about 15 days apart^37^. Therefore, this species is very suitable for exploring the genetic basis of individual variation in the timing of breeding behavior. Based on evidence from blue tits and barn swallows^23,26^, we hypothesized that individuals with longer *Clock* gene alleles initiate the HPG endocrine axis later, and consequently breed later than those with shorter *Clock* gene alleles. To test this hypothesis, we investigated the relationship between the secretion rate of plasma LH, T (males only), E_2_ (females only) and *Clock* gene poly-Q region length, in wild-caught birds housed in field aviaries at the study site in the spring of each year between 2014 and 2016. We also investigated the relationship between laying dates and nestling ClkpolyQ length in free-living birds at the study site. In this part of the study we inferred mean ClkpolyQ allele length of two parents from mean allele length of all their nestlings because breeding adults were difficult to catch, and because the mean allele length of parents would be expected to be equal to the mean allele length of all their offspring.

## Results

**Figure 1:**
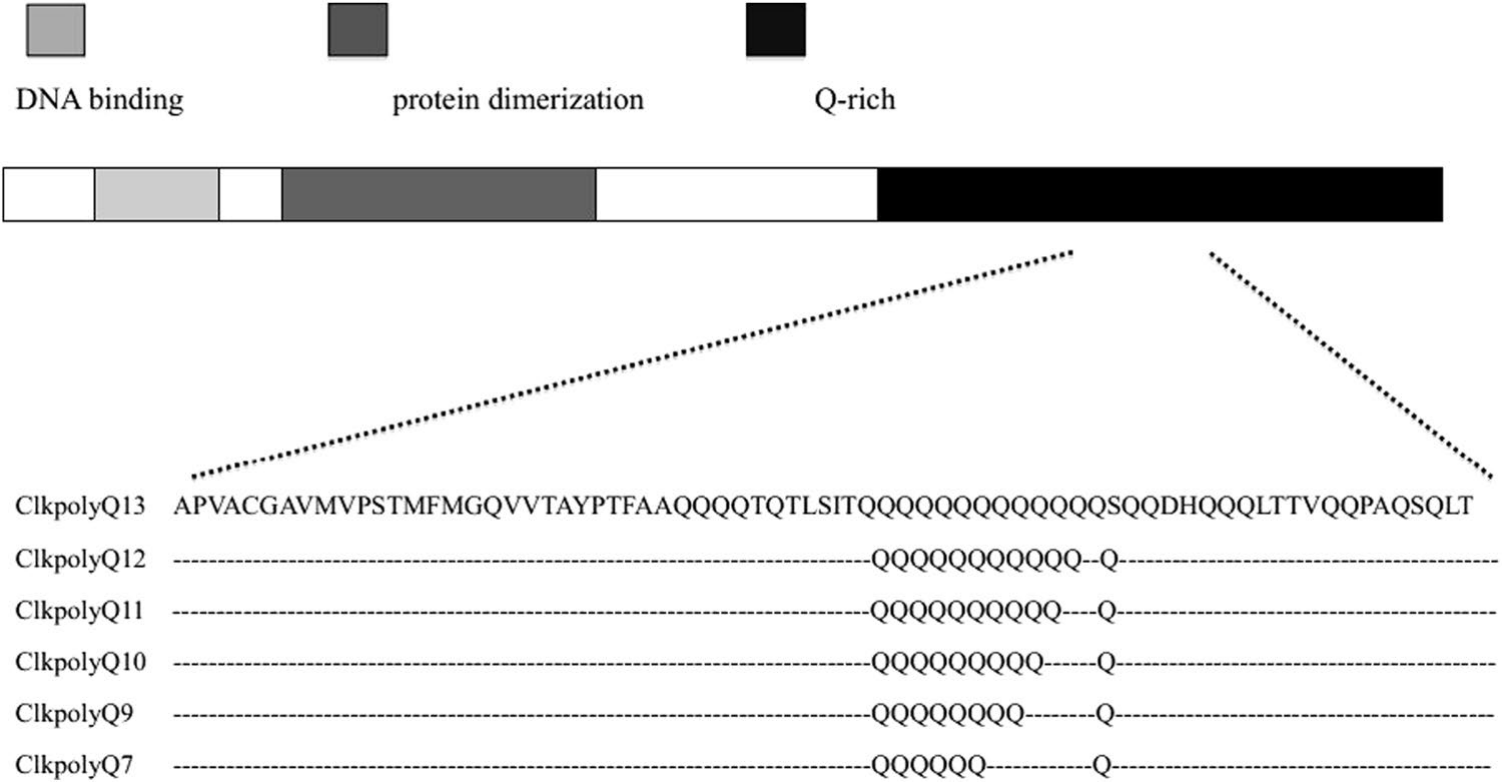
CLOCK poly-Q types identified in an Asian short-toed lark population in Inner Mongolia, China.

We successfully amplified, sequenced, and genotyped, the ClkpolyQ variable length region of all birds we collected blood samples from. Six length variants of ClkpolyQ alleles were identified: ClkpolyQ_7, 9, 10, 11, 12, 13_ (the subscript indicates the number of poly-Q repeats) (Fig. 1). Clkpoly Q_9,_ and ClkpolyQ_11_ were the two most common alleles (Table 1). *Clock* Poly-Q allele frequencies did not deviate significantly from the Hardy–Weinberg equilibrium (HWE). Observed overall heterozygosity is shown in Table 1.

**Table 1:** *Clock* gene poly-Q allele frequencies and observed heterozygosity (*H*) of adult Asian short-toed larks in Inner Mongolia, China.

	n	Q7	Q9	Q10	Q11	Q12	Q13	*H*
Total	257	0.004	0.438	0.050	0.434	0.041	0.033	0.585
Males (2014 + 2015 + 2016)	171	0.000	0.456	0.044	0.450	0.031	0.019	0.540
Females (2014 + 2015 + 2016)	86	0.012	0.402	0.061	0.402	0.061	0.061	0.674

**Figure 2:**
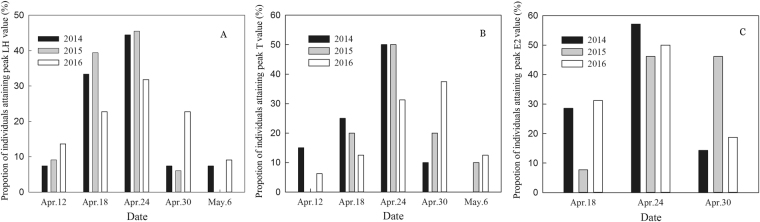
Variation in the dates on which (**a**) plasma LH, (**b**) T, and (**c**) E_2_, concentrations of individuals attaining the peak levels within a population of Asian short-toed larks in Inner Mongolia, China.

**Figure 3:**
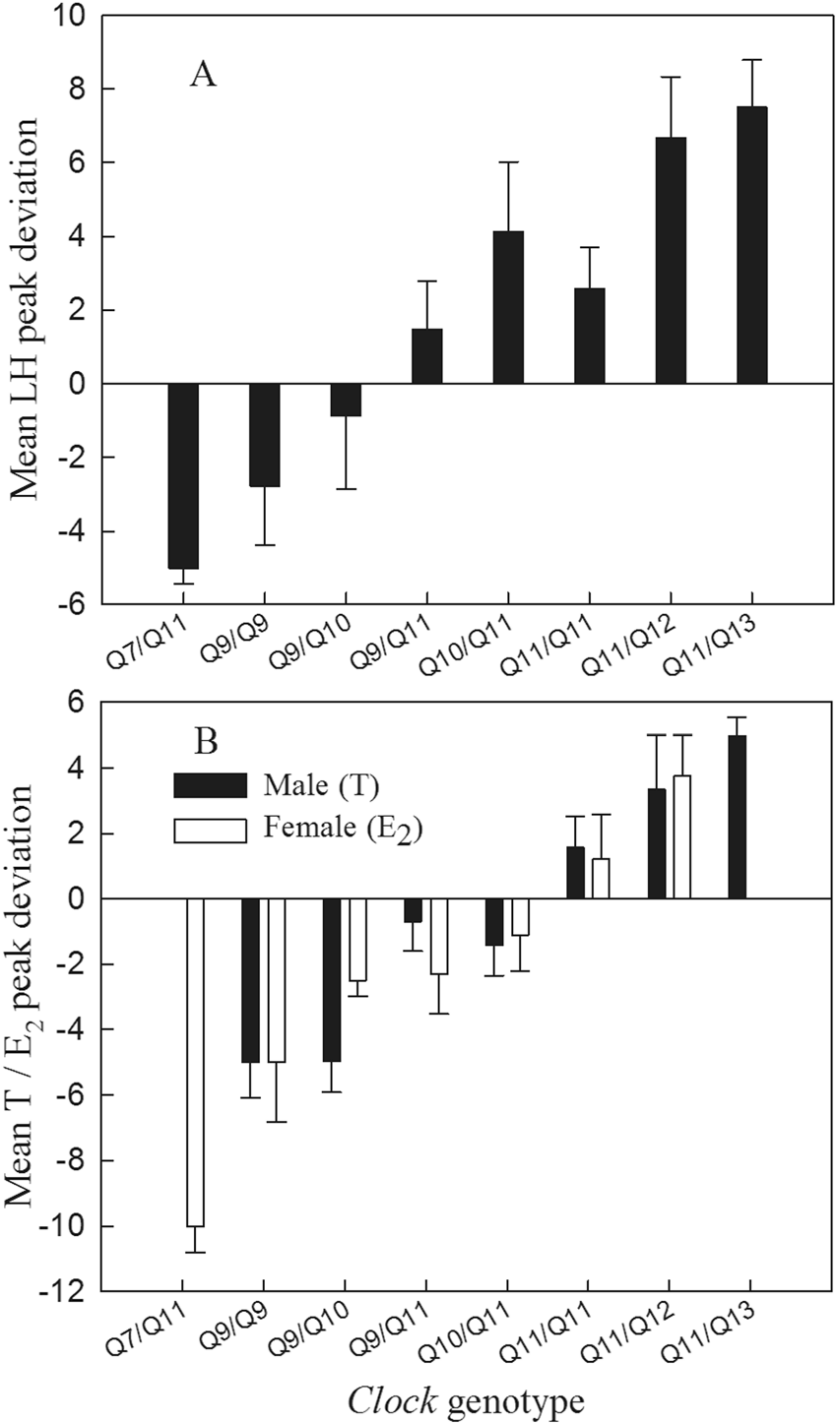
The LH, T and E_2_ mean peak date deviation of all *Clock* poly-Q genotypes. The mean LH, T and E_2_ peak date of the population in each year was scored as 0. The peak deviation of an individual bird was defined as the deviation days from the population peak date.

**Figure 4:**
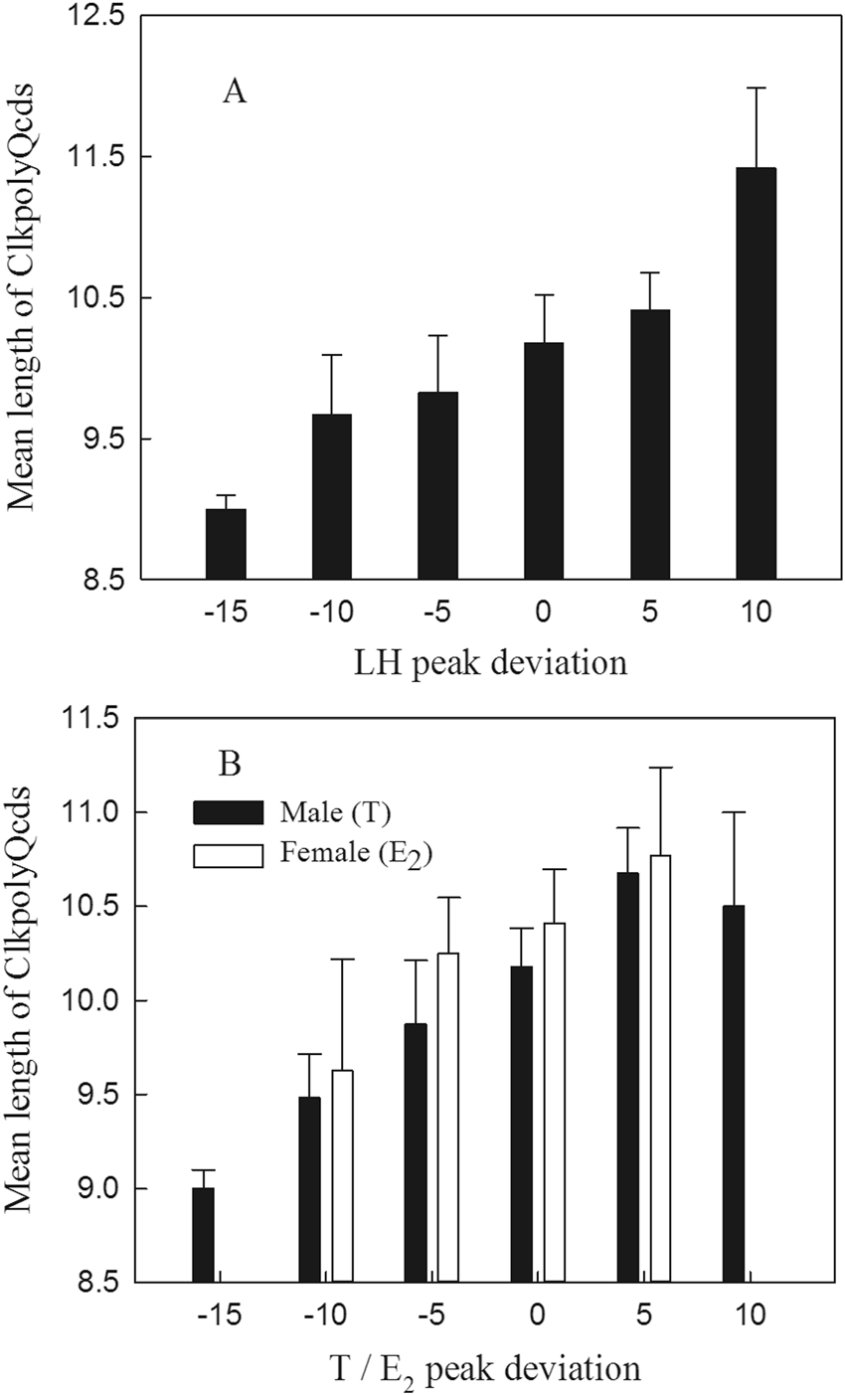
The mean lengths of *Clock* poly-Q allele length of the birds with different LH, T and E2 peak date deviation.

**Figure 5:**
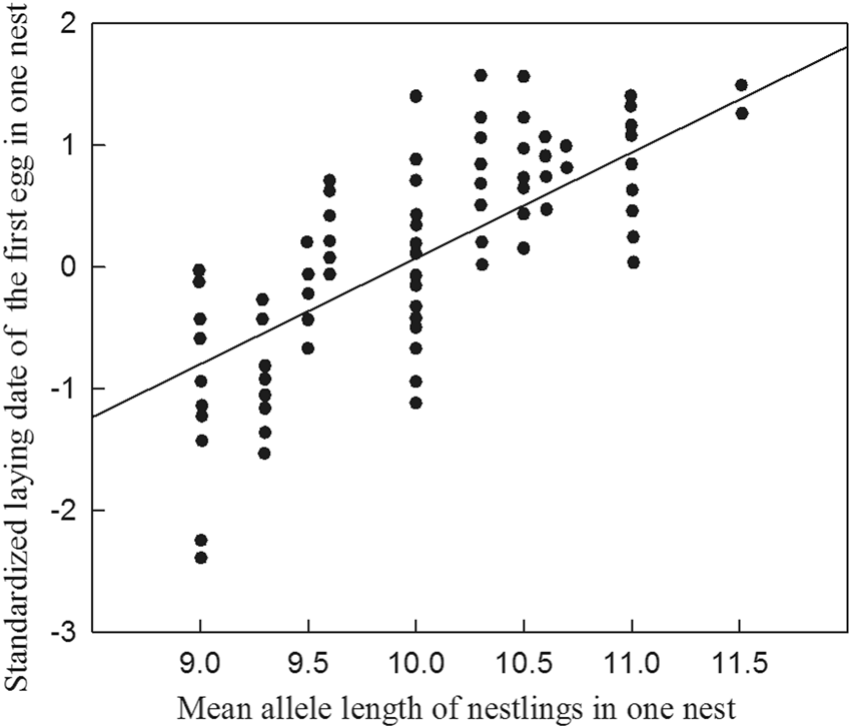
The correlation between mean *Clock* poly-Q allele length of the nestlings in one nest and the standardized laying date of the first egg in one nest (n = 240; r = 0.559; *P* < 0.001).

There was considerable individual variation in the dates on which plasma LH, T and E_2_ concentrations peaked in all three years of the study (Fig. 2). The results of LMMs indicate that of the explanatory variables ClkpolyQ length, body mass, sex and year, only ClkpolyQ length had a significant effect on plasma LH, T and E2 Peak date Deviation (PD; see Methods for definition) values (Table 2). Individual mean ClkpolyQ allele length (see Methods for definition) was positively correlated with the individual LH, T and E_2_ PD of adult birds (LH: n = 257, r = 0.541, *P* < 0.001; T: n = 171, r = 0.484; *P* < 0.001; E_2_: n = 86, r = 0.523, *P* = 0.001). The mean plasma LH, T and E2 PD of individuals with shorter ClkpolyQ genotypes were smaller (Fig. 3) and individuals with lower PD values had shorter mean allele lengths (Fig. 4). The mean ClkpolyQ allele length of nestlings in one nest was positively correlated with the first egg laying date for that nest (n = 240; r = 0.559; *P* < 0.001) (Fig. 5).Generally, the shorter a nestling’s mean ClkpolyQ allele length, the earlier the eggs were laid.

**Table 2:** Results of linear mixed models (LMMs) on the effects of ClkpolyQ length, body mass, sex, and year, on peak date deviation (PD) of plasma luteinizing hormone (LH), and on the PDs of plasma testosterone (T) and estradiol (E_2_), of wild-caught, captive, Asian short-toed larks in Inner Mongolia, China.

Response variable	Explanatory variable	F	*P*
PD of LH	ClkpolyQ length	15.907	<0.001
	body mass	0.509	0.826
	sex	0.888	0.348
	year	0.535	0.846
	ClkpolyQ length × body mass	0.535	0.846
	ClkpolyQ length × sex	1.819	0.167
	ClkpolyQ length × year	1.648	0.154
PD of T	ClkpolyQ length	3.333	**0**.**042**
	body mass	1.942	0.079
	year	0.982	0.473
	ClkpolyQ length × body mass	1.562	0.132
	ClkpolyQ length × year	0.971	0.516
PD of E_2_	ClkpolyQ length	4.482	**0**.**021**
	body mass	0.332	0.914
	year	1.025	0.431
	ClkpolyQ length × body mass	1.459	0.263
	ClkpolyQ length × year	1.042	0.348

## Discussion

Our results show that there is considerable within year individual variation in ClkpolyQ length in the Asian short-toed lark population, and that the ClkpolyQ length of individual birds is associated with temporal variation in the activation of reproductive hormone secretion and laying date. This supports our hypothesis that individuals with longer ClkpolyQ lengths activate the HPG axis and initiate breeding activity relatively late. Taken together, these results suggest that within year individual variation in the activation of the HPG axis and timing of breeding behavior in the Asian short-toed lark may have a genetic basis.

Although several studies have found evidence that the *Clock* gene influences the timing of laying in passerine birds^23,26^, the physiological mechanism responsible for this has not been identified. Our results imply that the *Clock* gene influences the timing of laying via the HPG axis. The discovery that the *Clock* gene may influence the timing of reproduction by binding to E-box elements in the GnRH receptor gene promoter of rats suggests that it regulates reproductive gene expression in the upper stream of the HPG axis^35^. Vitellogenesis and oocyte growth in birds are characterized by the estrogen-dependent hepatic synthesis of two main yolk precursors: VTG and yolk-targeted very low-density lipoprotein (VLDLy). These are the primary sources of yolk protein and lipid, respectively, providing all the nutrients and energy required by the developing embryo^38–40^. Estrogens initiate gene transcription and synthesis of VTG and apo-VLDL-II protein, and also reprogramme hepatic lipid and protein synthesis to support the transition from generic VLDL to VLDLy synthesis. Therefore, females in which E2 levels increase earlier could complete vitellogenesis sooner, and consequently lay earlier. Further research on the relationship between the *Clock* gene and the HPG axis in birds is required to clarify the physiological mechanism through which *Clock* affects the timing of breeding.

The majority of studies suggest that the timing of seasonal reproduction of birds is influenced by environmental cues and there is considerable knowledge of the potential mechanisms through which day length, ambient temperature and food availability affect the timing of reproduction^41–44^. However the potential mechanisms underlying individual variation in the timing of reproduction remain unclear. Our results reveal that such individual variation could have a genetic basis such that individuals with different *Clock* genotypes activate the HPG axis, and consequently breed, at different times. This suggests that the timing of reproduction in birds is not only influenced by environmental factors, but also by endogenous factors.

Long-term studies of wild bird populations have shown that the timing of reproduction is often under strong selection within populations and that females that time reproduction to coincide with peaks of food availability produce a greater proportion of the next generation^45,46^. It has been well established that the phenology of many species has been affected by global warming, which threatens the survival of some species^47,48^^.^ A trophic mismatch between birds and insects has been described in several European birds^49–52^. This is thought to be caused by the timing of reproduction in birds and the emergence of insects (the main food of nestlings) being initiated by different environmental cues^53,56^. Individual phenotypic polymorphism exists within populations, and if heritable, may allow populations to adapt to environmental change through microevolution. Physiologists are in general less interested in variation among individuals than in inter-species or inter-population variation^53–55^. Our results show that within year individual variation in the timing of reproductive hormone secretion and breeding behavior could have a genetic basis, and that this could be an important component of the microevolutionary adaptive potential of Asian short-toed lark populations. Asian short toed larks live on the grasslands of northeast China where the main food of nestlings (grasshoppers) peak at different times in different years due to variation in local ambient temperature^36^. As the timing of these annual food peaks in our study area varied significantly, polymorphism in the *Clock* gene could allow individual Asian short toed larks to breed successfully despite this marked annual fluctuation in peak nestling food abundance^36^. Further work is required to investigate how the Asian short-toed lark *Clock* gene might evolve in response to climate change.

## Methods

### Study site and species

The study site was located in the Dalai National Nature Reserve (47°45′50″N − 49°20′20″N; 116°50′10″E − 118°10′10″E) situated in the northeastern part of the Inner Mongolian Autonomous Region, China. This reserve is a semiarid, steppe region where the mean annual temperature, precipitation, and potential evaporation are −0.6 °C, 283 mm, and 1754 mm, respectively. The dominant plant species are *Stipa krylovii*, *Leymus chinensis*, and *Cleistogenes squarrosa*. Winter is longer than summer and the average maximum daytime temperatures in January and July are −20.02 °C and −22.72 °C, respectively. The Asian short-toed lark is the most common lark species on the grasslands of northeastern Inner Mongolia. This species begins nesting in the middle of April. Nest-building takes 7–10 days, the average clutch size is 3.05 ± 0.51, and the incubation period is 10–12 days^36^.

### Experiment design and data collection

Adult Asian short-toed larks were captured in mist-nets from March 10 to March 15 in 2014, 2015 and 2016 in the study site (sample sizes in each year are shown in Table 1) and were individually identified by leg-bands when first captured to avoid repeated sampling of the same individuals in different years. All captured birds were housed in out-door aviaries (100 × 100 × 70 cm) (six birds per aviary), fed mixed seeds, boiled eggs, and mealworms, and provided with water. From 29 March to 6 May, at least 100 μl of whole blood was collected from each bird between 12:00 and 12:30 every 5 days for LH, T, E_2_, and *Clock* gene, assays. Blood was collected into heparinized microcapillary tubes within 1–3 min of capture by puncturing a brachial wing vein with a disinfected 23 G needle. The skin around the puncture site was disinfected with medical alcohol before, and after, puncturing. Pressure was applied to the puncture site for 1 min with an alcohol-soaked cotton wool swab to stop bleeding. Blood samples were stored at 4 °C for up to 8 h until centrifuged at 3000 rpm for 10 min. The resultant blood plasma and cells were separated into different micro-centrifuge tubes and then kept frozen until assayed. Blood plasma was used for the hormone assays and the blood cells were used as a source of DNA to analyze *Clock* gene polymorphism. All caged birds were released at the end of the experiment. Experimental procedures comply with the ARRIVE guidelines and were carried out in accordance with the National Institutes of Health guide for the care and use of Laboratory animals (NIH Publications No. 8023, revised 1978), and approved by the Animal Research Ethics Committee of Hainan Provincial Education Centre for Ecology and Environment, Hainan Normal University (permit no. HNECEE-2013–002).

We started monitoring laying behavior of the free living birds on April 20 each year. Eighty, 82 and 78 nests were monitored from the first egg to fledging in 2014, 2015 and 2016, respectively. Laying dates of each egg were recorded for each nest. At least 20 μl of whole blood was collected from each six day old nestling for the *Clock* gene assay. The blood sampling and storage protocols for nestlings were the same as those for adults.

### Hormone assay

Plasma LH, T and E2 levels were measured using chicken enzyme immunoassay kits from MyBioSource (cat #MBS165746), Enzo (cat #ADI-901–065 and −174), respectively, which had been previously validated by our laboratory for use on Tree sparrows (*Passer montanus*)^56^. In order to confirm that the kits would work on Asian short-toed lark plasma, plasma samples from 20 Asian short-toed larks were pooled and diluted by 1, 1/2, 1/4, 1/8, 1/16 and 1/32, according to the methods used by Chastel *et al*.^57^. We then used the kits to analyze the concentration-dependent binding dynamics of the diluted samples. The resultant LH, T and E2 dilution curves were parallel to the standard ELISA Kit curves, confirming that the kits could reliably assess levels of LH, T and E2 in Asian short-toed lark plasma. The respective inter- and intra-plate coefficients of variation for LH, T and E_2_ were, 7.06% and 6.5%, 9.1% and 7.7%, and 8.5% and 2.4%, respectively.

### Sequencing of the *Clock* gene

We extracted total DNA from blood samples for PCR reactions^58^. PCR primers (forward primer: 5′-TTTTCTCAAGGTCAGCAGCTTGT-3′ and reverse primer: 5′-CTGTAGGAACTGTTG (C/T) GG (G/T) TGCTG-3′) were designed to amplify an approximately 250–290 bp region of the *Clock* gene for fragment-length analysis. The *Clock* gene was amplified using touchdown PCR in 25 μL reactions (12.5ulPremix (rTaq), 9.5ul dd H_2_O, 1ul of each primer (20pmol/ul), and 1ul template DNA (approximately 50–100ng/ul)) under the following conditions: 94 °C/2 min; 94 °C/30 s, 58 °C/30 s, 72 °C/60 s, 10 cycles; 94 °C/30 s, 62 °C/30 s, 72 °C/60 s increasing 5 s/cycle, 30 cycles; 72 °C/7 min, hold at 4 °C, catalyzed using Taq DNA polymerase. Amplified PCR products were prepared for sequencing using QIAGEN MinElute PCR Purification Kits. Nucleotide sequences of purified PCR fragments were determined with the BigDye Terminator version 3.1 cycle sequencing ready reaction (Applied Biosystems) under standard sequencing conditions according to the manufacturer’s protocol. The reaction products were detected on an ABI PRISM genetic analyser 3730xl (Applied Biosystems), and analyzed with the GeneMapper 4.0 software package. We succeeded in genotyping 99.6% of adult samples and 96% of nestling samples. After sequencing, we translated *Clock* gene sequences into *Clock* amino acid sequences with DNAMAN software, and counted the number of the Q in the poly-Q regions.

### Data analysis

We used “peak date deviation (PD) ” to measure the secretion rate of LH, T and E_2_. Firstly we calculated the mean LH, T and E_2_ values of all the birds on each sample day in each year. The dates on which the peak mean LH, T and E2 values occurred were defined as the peak dates for each hormone of the population in each year. The peak date of the population in each year was scored as 0. If the peak LH, T and E_2_ values of an individual bird occurred n days earlier than the population peak date, in which case the PD of the individual was recorded as −n. However, if the peak LH, T and E_2_ dates of an individual bird was n days later than the mean peak date, the PD of the individual was recorded as n.

Timing of laying of the free living birds were standardized for each year using the method described by Liedvogel *et al*.^26^. The laying date (LD) is defined as the number of days that the actual LD was from January 1^st^. Standardized laying date (LDstand) was defined as follows: LDstand = (LD-LD (average per year))/SD (where SD is the standard deviation of LD per year). Because a clutch can be laid within two days, we recorded the laying date of each clutch as the date on which the first egg was laid.

We defined *Clock* poly-Q (ClkpolyQ) genotype of an individual as the mean *Clock* poly-Q allele length, which allows the combined effect of both alleles on phenotype to be assessed. The mean poly-Q allele length of all nestlings in one nest was used to reflect the mean allele length of their parents.

The effects of ClkpolyQ length, body mass, adult sex, and sample year, on plasma LH PD were analyzed with a Linear Mixed Model (LMM) in which individual was a random factor. The effects of all factors, except sex, on the plasma T and E_2_ PD of adults were assessed with a LMM with individual as a random factor. We use Pearson correlation analysis to analyze the correlation between individual ClkpolyQ length and the PD of three hormones and the correlation between the mean ClkpolyQ length of the nestlings in one nest and the LD _stand_. Statistical analyses were performed in SPSS 18.0; α = 0.05 in all tests.

### Data availability

All data generated or analyzed during this study are included in this published article

## References

[1] Arnold TW (1992). Variation in laying date, clutch size, egg size, and egg composition of yellow-headed blackbirds (Xanthocephalus xanthocephalus): A supplemental feeding experiment. Can. J. Zool ..

[2] Camfield AF, Pearson SF, Martin K (2010). Life history variation between high and low elevation subspecies of horned lark Eremophila spp. J. Avian. Biol..

[3] Travers M, Clinchy LM, Boonstra R, Zanette L, Williams TD (2010). Indirect predator effects on clutch size and the cost of egg production. Ecol. Lett..

[4] van der Jeugd H, McCleery RH (2002). Effects of spatial autocorrelation, natal philopatry and phenotypic plasticity on the heritability of laying date. J. Evolution. Biol..

[5] McCleery RH (2004). Components of variance underlying fitness in a natural population of the great tit Parus major. Am. Nat..

[6] Sheldon BC, Kruuk LEB, Merilä J (2003). Natural selection and inheritance of breeding time and clutch size in the collared flycatcher. Evolution.

[7] Visser ME (2011). Genetic variation in cue sensitivity involved in avian timing of reproduction. Funct. Ecol..

[8] Nussey DH, Postma E, Gienapp P, Visser M (2005). Selection on heritable phenotypic plasticity in a wild bird population. Science.

[9] Hoekstra HE, Coyne JA (2007). The locus of evolution: evo devo and the genetics of adaptation. Evol. Int. J. Org. Evol..

[10] Ellegren H, Sheldon BC (2008). Genetic basis for fitness differences in natural populations. Nature.

[11] Johnsen A (2007). Avian Clock gene polymorphism: evidence for a latitudinal cline in allele frequencies. Mol. Ecol..

[12] Steinmeyer C, Mueller JC, Kempenaers B (2009). Search for informative polymorphisms in candidate genes: clock genes and circadian behaviour in blue tits. Genetica.

[13] Abzhanov A, Protas M, Grant BR, Grant PR, Tabin CJ (2004). Bmp4 and morphological variation of beaks in Darwin’s finches. Science.

[14] Fitzpatrick MJ (2005). Candidate genes for behavioural ecology. Trends. Ecol. Evol..

[15] Fidler AE (2007). DRD4 gene polymorphisms are associated with personality variation in a passerine bird. Proc. R. Soc. Lond. B..

[16] Bourret A, Garant D (2015). Candidate gene–environment interactions and their relationships with timing of breeding in a wild bird population. Ecol. Evol..

[17] Darlington TK (1998). Closing the circadian loop: CLOCK-induced transcription of its own inhibitors per and tim. Science.

[18] Johnsen A (2007). Avian clock gene polymorphism: evidence for a latitudinal cline in allele frequencies. Mol. Ecol..

[19] O’Malley KG, Banks MA (2008). A latitudinal cline in the Chinook salmon (Oncorhynchus tshawytscha) clock gene:evidence for selection on PolyQ length variants. Proc. Biol. Sci..

[20] Liedvogel M, Sheldon BC (2010). Low variability and absence of phenotypic correlates of Clock gene variation in a great tit Parus major population. J. Avian Biol..

[21] Dor R (2012). Clock gene variation in Tachycineta swallows. Ecol. Evol..

[22] Mueller JC, Pulido F, Kempenaers B (2011). Identification of a gene associated with avian migratory behaviour. Proc. Biol. Sci..

[23] Caprioli M (2012). Clock gene variation is associated with breeding phenology and maybe under directional selection in the migratory barn swallow. PLoS ONE.

[24] Liedvogel M, Cornwallis CK, Sheldon BC (2012). Integrating candidate gene and quantitative genetic approaches to understand variation in timing of breeding in wild tit populations. J. Evol. Biol..

[25] Peterson MP (2013). Variation in candidate genes CLOCK and ADCYAP1 does not consistently predict differences in migratory behavior in the songbird genus Junco. F1000Res..

[26] Liedvogel M, Szulkin M, Knowles SCL, Wood MJ, Sheldon BC (2009). Phenotypic correlates of *Clock* gene variation in a wild blue tit population: evidence for a role in seasonal timing of reproduction. Mol. Ecol..

[27] Van Noordwijk AJ, McCleery RH, Perrins CM (1995). Selection for the timing of Great Tit breeding in relation to caterpillar growth and temperature. J. Anim. Ecol..

[28] Naef-Daenzer B, Keller LF (1999). The foraging performance of Great and Blue tits (Parus major and P. caeruleus) in relation to caterpillar development, and its consequences for nestling growth and fledging weight. J. Anim. Ecol..

[29] Tremblay I, Thomas DW, Lambrechts M, Blondel MJ, Perret P (2003). Variation in Blue Tit breeding performance across gradients in habitat richness. Ecology.

[30] Thomas DW (2007). Common paths link food abundance and ectoparasite loads to physiological performance and recruitment in nestling Blue Tits. Funct. Ecol..

[31] Millar RP, King JA (1983). Synthesis and biological activity of [D-Trp6] chicken luteinizing hormone-releasing hormone. Peptides..

[32] Hattori A, Ishii S, Wada M (1986). Effects of two kinds of chicken luteinizing hormone-releasing hormone (LH-RH), mammalian LH-RH and its analogos on the release of LH and FSH in Japanese quail and chicken. Gen. Comp. Endocrinol..

[33] Lam F, Farner DS (1976). The ultrstructure of the cells of Leydig in the white-crowned sparrow (Zonotrichia leucophrys gambelii) in relation to plasma levels of luteinizing hormone and testosterone. Cell. Tissue. Res..

[34] Palmer SS, Bahr JM (1992). Follicle stimulating hormone increases serum oestradiol-17β concentrations, number of growing follicles and yolk deposition in aging hens (Gallus domessticus) with decreased egg production. Brit. Poultry. Sci..

[35] Resuehr D, Wildemann U, Sikes H, Olcese J (2007). E-box regulation of gonadotropin-releasing hormone (GnRH) receptor expression in immortalized gonadotrope cells. Mol. Cell.Endocrinol ..

[36] Zhang S (2017). Annual variation in the reproductive hormone and behavior rhythm in a population of Asian short toed lark: Can spring temperature influence activation of the HPG axis of wild birds?. Horm. Behav..

[37] Tian S, Wang W, Zhang S (2015). The breeding ecology of *Callendrella cheleensis* in Dalai Lake National Nature Reserve of Inner Mongolia. Sichuan Journal of Zoology.

[38] Rubin CJ (2010). Whole –genome resequencing reveals loci under selection during chicken domestication. Nature.

[39] Burley, R. W. & Vadehra, D.V. The Avian Egg: Chemistry and Biology. New York: John Wiley and Sons (1989).

[40] Walzem RL (1996). Lipoproteins and laying hen: Form follow function. Poult. Avian. Biol. Rev..

[41] Dawson A (2007). Seasonality in a temperate zone bird can be entrained by near equatorial photoperiods. Proc. Biol. Sci..

[42] Valle S, Carpentier, Elodie. Vu,B, Tsutsui K, Pierre D (2015). Food restriction negatively affects multiple levels of the reproductive axis in male house finches, Haemorhous mexicanus. J. Exp. Biol..

[43] Silverin B (2008). Ambient temperature effects on photo induced gonadal cycles and hormonal secretion patterns in great tits from three different breeding latitudes. Horm. Behav..

[44] Wingfield JC (2003). Effects of temperature on photoperiodically induced reproductive development, circulating plasma luteinizing hormone and thyroid hormones, body mass, fat deposition and molt in mountain white-crowned sparrows, Zonotrichia leucophrys oriantha. Gen. Comp. Endocrinol..

[45] Walzem RL, Hansen RJ, Williams DL, Hamilton RL (1999). Estrogen induction of VLDLy assembly in egg-laying hens. J. Nutr..

[46] Charmantier A (2008). Adaptive phenotypic plasticity in response to climate change in a wild bird population. Science.

[47] Sheldon BC, Kruuk LEB, Merila J (2003). Nature selection and inheritance of breeding time and clutch size in the collared flycatcher. Evolution.

[48] Parmesan C (2006). Ecological and evolutionary responses to recent climate change. Annu. Rev. Ecol.Evol. Syst..

[49] Hansen M, Olivieri I, Waller D, Nielsen E, Ge M (2012). Monitoring adaptive genetic responses to environmental change. Mol. Ecol..

[50] Visser ME, Holleman L, Gienapp P (2006). Shifts in caterpillar biomass phenology due to climate change and its impact on the breeding biology of an insectivorous bird. Oecologia..

[51] Cotton P (2003). Avian migration phenology and globalclimate change. Proc. Natl. Acad. Sci. USA.

[52] Both C, Bouwhuis S, Lessells C, Visser M (2006). Climate change and population declines in a long-distance migratory bird. Nature.

[53] Both C (2010). Avian population consequences of climate change are most severe for long-distance migrants in seasonal habitats. Proc. Biol. Sci..

[54] Miles JE, Bale JS, Hodkinson ID (1997). Effects of temperature elevation on the population dynamics of the upland heather psyllid Strophingia ericae (Curtis) (Homoptera: Psylloidea). Global. Change. Biol..

[55] Ball G, Balthazart J (2008). Individual variation and the endocrine regulation of behavior and physiology in birds: a cellular/molecular perspective. Phil. Trans. R. Soc. B..

[56] Zhang S, Chen X, Zhang J, Li H (2014). Differences in the reproductive hormone rhythm of tree sparrows (Passer montanus) from urban and rural sites in Beijing: The effect of anthropogenic light sources. Gen. Comp. Endocr..

[57] Chastel O (2005). High levels of LH and testosterone in a tropical seabird with an elaborate courtship display. Gen. Comp. Endocr..

[58] Griffith SC, Stewart IRK, Dawson DA, Owens IPF, Terry B (1999). Contrasting levels of extra-pair paternity in mainland and island populations of the house sparrow (*Passer domesticus*): is there an “island effect”?. Biol. J. Linn. Soc..

